# Subunit Vaccine ESAT-6:c-di-AMP Delivered by Intranasal Route Elicits Immune Responses and Protects Against *Mycobacterium tuberculosis* Infection

**DOI:** 10.3389/fcimb.2021.647220

**Published:** 2021-03-22

**Authors:** Huanhuan Ning, Wei Zhang, Jian Kang, Tianbing Ding, Xuan Liang, Yanzhi Lu, Chengxuan Guo, Wenjie Sun, Huapeng Wang, Yinlan Bai, Lixin Shen

**Affiliations:** ^1^ Key Laboratory of Resources Biology and Biotechnology in Western China, College of Life Sciences, Northwest University, Xi’an, China; ^2^ Department of Microbiology and Pathogen Biology, Basic Medical School, Air Force Medical University, Xi’an, China; ^3^ Department of Paediatrics, TangDu Hospital, Air Force Medical University, Xi’an, China; ^4^ Medical College, Xijing University, Xi’an, China; ^5^ Student Brigade, Basic Medical School, Air Force Medical University, Xi’an, China

**Keywords:** *Mycobacterium tuberculosis*, subunit vaccine, ESAT-6, c-di-AMP, mucosal adjuvant

## Abstract

Tuberculosis (TB), caused by *Mycobacterium tuberculosis* (Mtb) infection, remains the most common cause of death from a single infectious disease. More safe and effective vaccines are necessary for preventing the prevalence of TB. In this study, a subunit vaccine of ESAT-6 formulated with c-di-AMP (ESAT-6:c-di-AMP) promoted mucosal and systemic immune responses in spleen and lung. ESAT-6:c-di-AMP inhibited the differentiations of CD8^+^ T cells as well as macrophages, but promoted the differentiations of ILCs in lung. The co-stimulation also enhanced inflammatory cytokines production in MH-S cells. It was first revealed that ESAT-6 and c-di-AMP regulated autophagy of macrophages in different stages, which together resulted in the inhibition of Mtb growth in macrophages during early infection. After Mtb infection, the level of ESAT-6-specific immune responses induced by ESAT-6:c-di-AMP dropped sharply. Finally, inoculation of ESAT-6:c-di-AMP led to significant reduction of bacterial burdens in lungs and spleens of immunized mice. Our results demonstrated that subunit vaccine ESAT-6:c-di-AMP could elicit innate and adaptive immune responses which provided protection against Mtb challenge, and c-di-AMP as a mucosal adjuvant could enhance immunogenicity of antigen, especially for innate immunity, which might be used for new mucosal vaccine against TB.

## Introduction

Tuberculosis (TB) remains the most common cause of death from a single infectious disease. Approximately two billion people worldwide are infected with *Mycobacterium tuberculosis* (Mtb), with around 10 million new cases of TB emerging each year and approximately 1.4 million deaths in 2019 (WHO, 2020). Bacillus Calmette-Guerin (BCG) is the only licensed TB vaccine, and provides effective protection against TB meningitis and miliary TB when inoculated *via* the intradermal route in children, but its efficacy is variable against pulmonary diseases in adults and does not confer long-lasting protection (Mangtani et al., 2014). As of 2020, there are 14 new TB vaccines in clinical trials, and four of those are subunit vaccines composed of serial Mtb antigens with different adjuvants (WHO, 2020). Subunit vaccine exhibits superior safety and activates stronger antigens-specific immune response compared with other vaccines such as DNA vaccine and attenuated live mycobacterial vaccine (Zhu et al., 2018). More efforts are being made in the formulation, adjuvants, and delivery methods of subunit vaccine to improve the protection against Mtb infection.

The 6 kDa early secretory antigenic target (ESAT-6), an abundantly secreted protein identified from the secreted culture filtrate of Mtb, is a promising candidate antigen for subunit vaccine (Unnikrishnan et al., 2017). ESAT-6 is encoded by *esxA*, a gene in a genetic locus known as region of difference 1 (RD1), which is absent in BCG (Abdallah et al., 2007). Until now, two subunit vaccines composed of ESAT-6, GamTBvac (containing Ag85a and ESAT6-CFP10) and H56:IC31 (containing Ag85B, ESAT-6 and Rv2660c), are now being tested in Phase IIa and IIb trails respectively (WHO, 2020). Our previous work showed that fusion protein Ag85B-ESAT-6 adjuvanted with monophosphoryl lipid A (MPLA) induces significant humoral and cellular immune response by subcutaneous (s.c.) vaccination (Xu, 2014). While, ESAT-6 alone exhibits insufficient immunogenicity either immunized by s.c. or intramuscular (i.m.) route (Lu et al., 2018).

It is widely agreed that the mucosal immune response is vital for protecting against respiratory pathogens including Mtb (Copland et al., 2018; Paquin-Proulx et al., 2018). Mucosal vaccination leads to both mucosal and systemic responses due to dendritic cells (DCs) carrying the immunized antigen to systemic inductive sites such as the lymph nodes and spleen (Macpherson et al., 2008; Lycke, 2012). Several successful mucosal vaccines against diseases have been widely used, indicating the feasibility and safety of this approach. Vaccines against polio, cholera, rotavirus, as well as salmonella are administrated through the oral route (Stylianou et al., 2019). It was also found that intranasal (i.n.) administration of ESAT-6-CFP-10 was inclined to reinforcement of cellular immune responses than that of by i.m. and s.c. immunization (Namvarpour et al., 2019). Hence, mucosal vaccination of ESAT-6 with safe adjuvant may provide improved immune responses against Mtb.

Currently, there is no approved safe and reliable mucosal adjuvant for subunit vaccine. Cyclic dimeric adenosine monophosphate (c-di-AMP), a bacterial second messenger, regulates the cellular physiologies including bacterial growth, biofilm formation, potassium homeostasis, fatty acid metabolism, and virulence (Devaux et al., 2018; Commichau et al., 2019; Zarrella and Bai, 2020). Moreover, c-di-AMP from bacteria activates the cytosolic surveillance pathway leading to the induction of type I interferons (IFNs) during infection (Woodward et al., 2010; Yang et al., 2014; Dey et al., 2015), which is mediated by the stimulator of interferon genes (STING) (Burdette et al., 2011). c-di-AMP derived from Mtb elicits increased autophagy, which restricts the intracellular bacteria growth (Dey et al., 2015).

Additionally, both model antigens β-galactosidase and ovalbumin co-administrated with c-di-AMP induced antigen-specific secreted IgA (sIgA) and balanced Th1/Th2/Th17 response pattern (Ebensen et al., 2011; Skrnjug et al., 2014). Pathogen specific antigens with c-di-AMP as a mucosal adjuvant conferred protection against influenza virus (Sanchez et al., 2014) or *Trypanosoma cruzi* (Matos et al., 2017). Our previous work demonstrated that c-di-AMP as endogenous adjuvant of recombinant BCG (rBCG) induced stronger immune responses in mice after Mtb infection, which was related to trained immunity (Ning et al., 2019). Another report of this rBCG enhanced the protective efficacy against TB in a guinea pig model (Dey et al., 2020). Thus, c-di-AMP exhibits a promising potential as an adjuvant for the development of subunit vaccines, especially for mucosal inoculation.

In this study, a subunit vaccine of ESAT-6 with c-di-AMP as an adjuvant (ESAT-6:c-di-AMP) was administrated by intranasal route *in vivo*, as well as macrophages *in vitro*, to be evaluated for its immune properties and protection against Mtb infection.

## Materials and Methods

### Bacteria Strains, Cell Lines, and Animals

*Mycobacterium tuberculosis* H37Ra were obtained from National Institute for Food and Drug Control (China) and grown in Middlebrook 7H9 medium (BD) supplemented with 10% oleic acid-albumin-dextrose-catalase (OADC) (BD) and 0.05% Tween 80, or 7H10 medium (BD) for plate. Murine alveolar macrophage cell line MH-S and monocyte/macrophages RAW 264.7 cells were purchased from Procell Life Science & Technology Co., Ltd. (China). SPF mice were purchased from Animal Center of Air Force Medical University.

### Animal Groups, Immunization, and Infection

Female BALB/c mice aged from 6 to 8 weeks were anesthetized and treated by intranasal immunization in 50 µl PBS containing c-di-AMP (5 μg/mouse), ESAT-6 (30 μg/mouse), or ESAT-6 (30 μg/mouse) with c-di-AMP (5 μg/mouse) for three times at 2-week intervals. ESAT-6 dose was reduced to 15 µg/mouse in the third immunization. PBS (50 µl/mouse) were used as a control [Naïve and un-vaccinated (UN)]. After 4-week immunization, mice were challenged with 5 × 10^4^ CFU of Mtb H37Ra intravenously in 100 μl PBS.

### Detection Antibodies by ELISA

Antibodies in sera and bronchoalveolar lavage fluid (BALF) were detected by enzyme-linked immunosorbent assay (ELISA). Recombinant ESAT-6 was coated to 96-well plate according to the procedures in our previous work (Lu et al., 2018). HRP-conjugated goat anti-mouse IgG, IgG1, IgG2a, IgG2b, IgG3, and IgA were used, respectively, as secondary antibodies. Subsequently, 3,3′,5,5′-tetramethylbenzidine (TMB) substrate solution was added for detection. The absorbance was determined at an optical density of 450 nm (OD_450_) using microplate reader (BioTek).

### Preparation of Single Cell Suspension From Lung

Lung tissues were cut into small pieces with sterilized scissors on ice, and then digested in 3 ml digestion media [RPMI 1640 media containing 5% fetal bovine serum (FBS) with 50 μg/ml DNase I (Sigma), 1 mg/ml collagenase V (Sigma), 100 U/ml penicillin, and 100 μg/ml streptomycin (Solarbio)] for 1h at 37°C with 5% CO_2_. The digested suspension was passed through 70 μm cell strainer and pelleted by centrifugation, then erythrocytes were lysed through osmotic shock buffer (150 mM NH_4_Cl, 10 mM KHCO_3_, and 0.1 mM EDTA, pH 7.2) for 1 min at room temperature. Cells were resuspended and adjusted to the appropriate densities for use.

### Measurement of Splenocytes Proliferation

Mouse spleen was aseptically removed and homogenized with 40 μm cell strainers. Single cell suspension was prepared as our previous work (Ning et al., 2019). Splenocytes were seeded in 96-well microplates at 2 × 10^5^ cells/well and stimulated with 5 μg/ml ESAT-6 at the indicated timepoints. MTS reagent was then added and incubated for another 3 h. The absorbance was determined at OD_490_ using microplate reader.

### Flow Cytometry

Cells were resuspended in 100 μl PBS containing viability stain Zombie NIR dye (BioLegend) and incubated for 20 min at room temperature in the dark. To avoid unspecific antibody binding, Fc receptors were blocked by anti-mouse CD16/32 mAb in Cell Staining Buffer (BioLegend) for 15 min on ice. Cells were then incubated in 100 μl Cell Staining Buffer with fluorochrome-conjugated cell surface antibodies for 15 min on ice shielded from light. Eventually, cells were washed and resuspended in 500 μl of Cell Staining Buffer for flow cytometry detection.

Intracellular cytokine staining was performed after surface molecule staining as described above. Cells were stimulated with ESAT-6 for 72 h and protein transport inhibitors Brefeldin A Solution (BioLegend) were added to culture in the last 12 h of stimulation prior to harvest. Cells were fixed and permeabilized according to the instructions of Cytofix/Cytoperm Fixation/Permeabilization Kit (BD). Then cells were resuspended in 50 μl of BD Perm/Wash buffer (BD) containing fluorochrome-conjugated antibodies and incubated at 4°C for 30 min in the dark. Finally, cells were washed with Perm/Wash buffer and resuspend in Staining Buffer for flow cytometric analysis. All labeled antibodies were listed in **Table S1**.

Flow cytometry was performed using a BD FACSAria and analyzed with FlowJo software version 10.0 (TreeStar, Ashland).

### qRT-PCR Analysis

Total RNA was extracted by TRIZol reagent (TaKaRa) according to the manufacturer’s instruction. cDNA was obtained by reverse transcription of 500 ng total RNA using PrimeScript RT reagent Kit with gDNA Eraser (TaKaRa). The primers for qRT-PCR synthesized by Tsingke Biological Technology of China. The sequences of primers were listed in **Table S2**. The fold change of target gene transcription was calculated by 2^−ΔΔCt^.

### Cytokine Secretion Assays

Splenocytes were seeded in 96-well microplate 1 × 10^6^ cells/well and stimulated with 5 μg/ml ESAT-6 protein for 72 h. Cell supernatants were collected to measure cytokine secretion by mouse ELISA kits for IFN-γ, IL-2, IL-10, IL-17, IL-1β, IL-18, IL-6, and TNF-α (eBioscience) according to the manufacturer’s instructions.

### Generation of Bone Marrow-Derived Macrophages (BMDMs)

Bone marrow from both femurs and tibiae of mice was harvested in RPMI 1640. Cells were subsequently resuspended in RPMI 1640 supplemented with 15% FBS, 100 U/ml penicillin, 100 μg/ml streptomycin, and 25% L929-conditioned media in petri dishes (100 mm) and incubated for 3 d at 37°C with 5% CO_2_, replaced the medium with the same fresh media. Cells were cultured for another 2 days allowing to differentiation. At day 5, cells were removed using 0.25% trypsin supplemented with 0.02% EDTA and cell scraper. Cells were harvested and resuspended in complete RPMI 1640 medium for *in vitro* assays.

### Stimulation of Macrophage Cell Line *In Vitro*

Mouse alveolar macrophage line MH-S cells, RAW264.7 macrophages, and BMDMs were seeded in six-well plates at 1 × 10^6^ cells/well in complete RPMI 1640 medium with Penicillin and Streptomycin solution and incubated overnight. Cells were stimulated by adding c-di-AMP and ESAT-6 with concentration and the duration of stimulation shown in the corresponding figure legends (Jung et al., 2017; Rueckert et al., 2017).

### Western Blot Analysis

Cells were lysed using RIPA buffer (Solarbio) supplemented with protease inhibitor cocktail complete (Roche) and phosphatase inhibitor cocktail (Roche) after treatment *in vitro*. LC3 (Sigma) and p62/SQSTM (Proteintech) antibodies were incubated respectively, β-actin (Proteintech) was detected as an internal reference protein.

### Immunofluorescence

Cells were fixed with 4% paraformaldehyde and permeabilized with 0.5% Triton X-100 and blocked with 3% BSA. LC3 puncta were stained with LC3 antibody, detected by FITC conjugated goat anti-rabbit antibody. Cell nucleus were stained with Hoechst 33342 for observation under Olympus fluorescence microscope. Extent of autophagy inductions were represented by the percentages of LC3 aggregates puncta-positive cells referring to previous study (Dey et al., 2015).

### Bacteria Survival in Macrophages

MH-S cells were stimulated by adding c-di-AMP (10 μg/ml) referred to the previous study (Rueckert et al., 2017), ESAT-6 (10 μg/ml), or ESAT-6 combined with c-di-AMP for 24 h. Log-phase cultures of Mtb H37Ra was washed and diluted in antibiotic-free RPMI, and then were added to the cells with a multiplicity of infection (MOI) at 2:1 for 4 h. The extracellular bacteria were removed by washing with sterile PBS three times, and this time point marked as “0 h” post infection. After infection, cells were washed thoroughly with PBS and lysed by 0.025% SDS at indicated time points. Lysis solutions were diluted and spread on 7H10 agar plates for 3-week of incubation at 37°C for bacteria CFUs counting.

### CFU Enumeration

After Mtb challenge, mice spleens and lungs were aseptically removed and homogenized with 40 μm strainer. Serial dilutions of tissue homogenates were spread on 7H10 agar plates, and CFUs were numerated after 3-week of incubation at 37°C.

### Statistical Analysis

Statistical analysis was performed using Graph Pad Prism 5.0. All measurements were replicated at least three times and the results expressed as mean ± SEM. The variance differences were compared by Student’s *t*-test or for multiple comparisons by analysis of variance (ANOVA). Significant differences were established if *P* < 0.05. “*/^#^” *P *< 0.05, “**/^##^” *P* < 0.01, “***/^###^” *P* < 0.001.

## Results

### ESAT-6:c-di-AMP Induced Systemic IgG and Higher Local Mucosal sIgA

**Figure 1:**
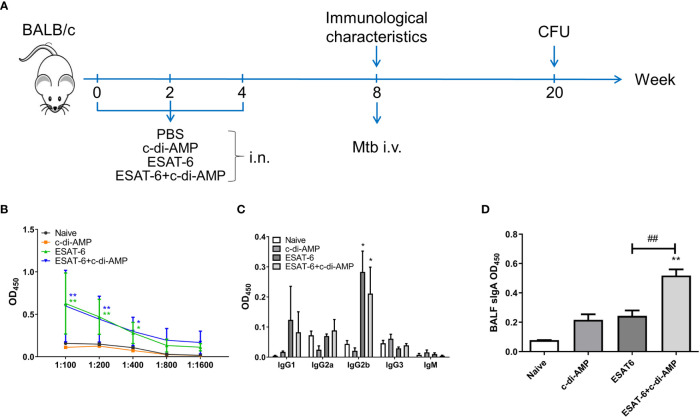
The subunit vaccine ESAT-6:c-di-AMP induced systemic IgG and enhanced mucosal sIgA. **(A)** The immunization and Mtb infection strategy scheme. Female BALB/c mice were immunized intranasally (i.n.) with PBS (Naïve group), c-di-AMP, ESAT-6, or ESAT-6 co-administrated with c-di-AMP, respectively. Mice were boosted twice at 2-week internals. Four weeks after the last immunization, mice were challenged intravenously (i.v.) with Mtb H37Ra at 5 × 10^4^ CFU. **(B)** ESAT-6-specific total IgG in sera at a series of dilutions from 1:100 to 1:1 600. **(C)** ESAT-6-specific subclasses of antibody in sera (1:200) of immunized mice. **(D)** ESAT-6-specific sIgA in BALF of immunized mice were determined using ELISA. “*,” compared with the control group (Naïve). *P<0.05, **^/##^P<0.01, ***P<0.001.

Our previous work found that ESAT-6 specific antibodies were relatively low in both Mtb-infected mice and guinea pig, which exhibited poor immunogenicity (Lu et al., 2018). Anti-ESAT-6 antibody titer exhibited only 1.13-fold higher in sera of TB patients than healthy control (**Figure S1**). In this study, mice were vaccinated with ESAT-6:c-di-AMP by i.n. route, and the immunization strategy scheme was shown in **Figure 1A**. ESAT-6 alone induced significant elevated total IgG response than Naïve mice (*P* < 0.01) (**Figure 1B**). ESAT-6:c-di-AMP vaccination induced comparable IgG with the antigen alone (**Figure 1B**). For antibody isotypes, either ESAT-6:c-di-AMP or ESAT-6 alone could induce increased IgG2b (**Figure 1C**). Humoral immune responses in the local mucosa are mainly mediated by sIgA, which is considered the hallmark antibody (Stylianou et al., 2019). ESAT-6 alone did not induce significant alteration of sIgA compared with Naïve mice in BALF (*P* > 0.05). Noticeably, c-di-AMP enhanced local mucosal sIgA secretion in BALF induced by ESAT-6 compared to that detected from Naïve mice (*P* < 0.01) (**Figure 1D**). These results indicated that subunit vaccine ESAT-6:c-di-AMP induces high systemic IgG and higher local mucosal sIgA, and c-di-AMP as a mucosal adjuvant enhances humoral immune response induced by ESAT-6.

### ESAT-6:c-di-AMP Promoted Th1/Th2/Th17 Immune Responses and Inflammatory Cytokines in Spleen

**Figure 2:**
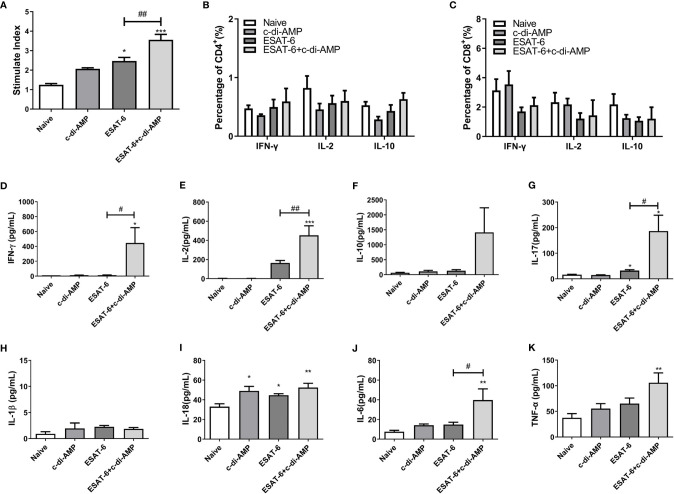
The subunit vaccine ESAT-6:c-di-AMP elicited stronger cellular responses in spleen. **(A)** Splenocyte proliferation of immunized mice stimulated by ESAT-6 (5 μg/ml) *in vitro*. IFN-γ, IL-2, IL-10 secreting splenocytes of CD4^+^ **(B)** and CD8^+^ **(C)** T cells detected by FCM after stimulated with ESAT-6 (5 μg/ml) *in vitro*. IFN-γ **(D)**, IL-2 **(E)**, IL-10 **(F)**, IL-17 **(G)**, IL-1β **(H)**, IL-18 **(I)**, IL-6 **(J)**, and TNF-α **(K)** release in supernatant of splenocytes detected using ELISA after stimulated with ESAT-6 (5 μg/ml) *in vitro*. “*,” compared with the control group (Naïve) *^/#^P<0.05, **^/##^P<0.01, ***P<0.001.

Cytokines play a crucial role both in controlling initial infection and in promoting as well as maintaining adaptive T-cell responses that mediate host resistance to pathogen (Shaw et al., 2018). Our data showed that immunization of mice with ESAT-6 alone stimulated 2.0-fold splenocytes proliferation than the Naïve group (**Figure 2A**), and c-di-AMP enhanced the proliferation induced by ESAT-6 (*P* < 0.01) (**Figure 2A**). It has been reported that c-di-AMP as a mucosal adjuvant enhances Th1/Th2/Th17 responses of antigen in mice (Ebensen et al., 2011). In this study, we did not find differences in the proportions of CD4^+^ and CD8^+^ T cells secreting IFN-γ, IL-2, and IL-10 between different groups after 72 h stimulation *in vitro* (**Figures 2B, C** **)**. Splenocytes from mice immunized with ESAT-6 alone produced increased IL-2 (*P* < 0.05) and IL-17 (*P* < 0.05) than Naïve mice (**Figures 2E, G** **)**. Moreover, c-di-AMP enhanced the secretions of IFN-γ (*P* < 0.05), IL-2 (*P* < 0.01), and IL-17 (*P* < 0.05) in splenocytes of ESAT-6:c-di-AMP immunized mice compared to the vaccination with ESAT-6 alone (**Figures 2D–G**).

In our previous work we found that rBCG with elevated c-di-AMP induced more cytokines related to trained immunity such as IL-1β, IL-6, and TNF-α in splenocytes (Ning et al., 2019). Both IL-1 and IL-18 belong to the interleukin-1 family of cytokines and were secreted following inflammasome activation, which involved in innate and adaptive immune system (Mantovani et al., 2019). It was showed that ESAT-6:c-di-AMP inoculation had no effect on IL-1β (**Figure 2H**), and slightly stimulated IL-18 secretion (**Figure 2I**) (*P* < 0.01). However, we found that ESAT-6:c-di-AMP induced highest levels of IL-6 (*P* < 0.01) and TNF-α (*P* < 0.01) compared to the control mice (**Figures 2H–K**). Overall, ESAT-6:c-di-AMP could potently induce the Th1/Th2/Th17 cellular immune responses and inflammatory cytokines release in spleen by mucosal inoculation, indicating a predictive protection against Mtb infection.

### ESAT-6:c-di-AMP Promoted Cytokine Responses in Lung

**Figure 3:**
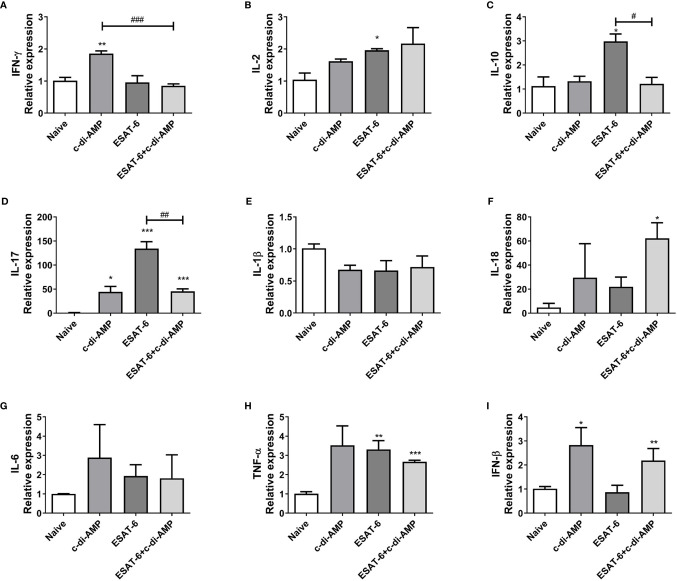
Subunit vaccine ESAT-6:c-di-AMP induced cytokines response in lung. mRNA levels of IFN-γ **(A)**, IL-2 **(B)**, IL-10 **(C)**, IL-17 **(D)**, IL-1β **(E)**, IL-18 **(F)**, IL-6 **(G)**, TNF-α **(H)**, and IFN-β **(I)** in lungs of immunized mice were examined using qRT-PCR. “*,” compared with the control group (Naïve) */^#^P<0.05, **/^##^P<0.01, ***/^###^P<0.001.

We showed that ESAT-6:c-di-AMP promoted Th1/Th2/Th17 as well as inflammatory cytokines response systematically in spleen (**Figure 2** **)**. Mice inoculated with ESAT-6:c-di-AMP also displayed similar mRNA levels of IFN-γ and IL-2 but decreased IL-10 (*P* < 0.05) and IL-17 (*P* < 0.01) in lungs compared to those vaccinated with ESAT-6 alone (**Figures 3A–D**), which suggested that c-di-AMP prevented increases of IL-10 and IL-17 in lung. For inflammatory cytokines, ESAT-6:c-di-AMP inoculation resulted in elevated IL-18 (*P* < 0.05) and TNF-α (*P* < 0.01) mRNA levels in lung than those in Naïve mice, but not significantly different from the group vaccinated with ESAT-6 alone (**Figures 3E–I** **)**. IFN-β levels were induced mainly by c-di-AMP in ESAT-6:c-di-AMP immunization group (*P* < 0.01), consistent with studies of c-di-AMP on IFN-β response in macrophages (Dey et al., 2015; Rueckert et al., 2017; Ning et al., 2019). Taken together, inoculation ESAT-6:c-di-AMP elicited Th17 and inflammatory cytokine responses in lung. ESAT-6 and c-di-AMP in subunit vaccine played respective roles through distinct mechanisms on immune cells in lung, which were different from that in spleen.

### ESAT-6:c-di-AMP Inhibited the Differentiation of CD8^+^T Cells in Lung

**Figure 4:**
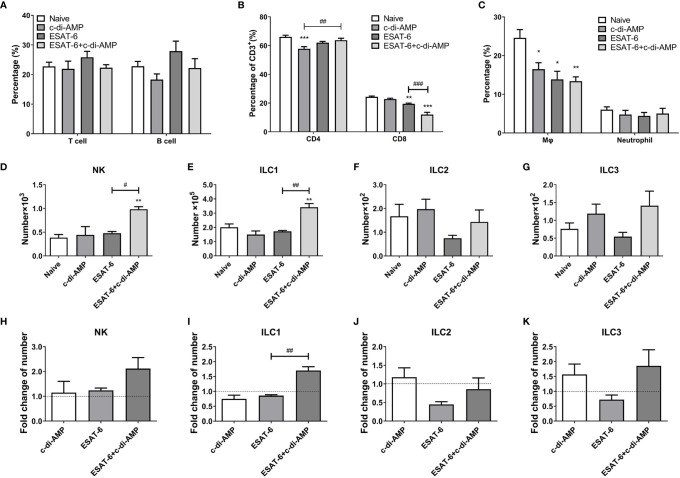
Subunit vaccine ESAT-6:c-di-AMP inoculation affected the differentiations of cell subsets in lung. Proportions of T and B cells **(A)**, CD4^+^ and CD8^+^ T cells **(B)**, and macrophages and neutrophils **(C)** in lungs of immunized mice. ILCs numbers of NK cells **(D)**, ILC1 **(E)**, ILC2 **(F)**, and ILC3 **(G)** in lungs of immunized mice 4-week after final immunization. **(H–K)** Fold changes of ILCs cells number related to Naïve mice in **(D–G)**. “*,” compared with the control group (Naïve) */^#^P<0.05, **/^##^P<0.01, ***/^###^P<0.001.

Further, the proportions of cell subsets in lungs were detected by FCM. ESAT-6 tended to induce cell proliferation of T and B cells, though no difference among all the groups (*P* > 0.05) (**Figure 4A**). To our surprise, it showed that c-di-AMP alone significantly inhibited CD4^+^ T cells (*P* < 0.001), and ESAT-6 inhibited CD8^+^ T cells (*P* < 0.01) in lung compared with Naïve group (**Figure 4B**). In contrast, the decline of CD8^+^ T cells induced by ESAT-6 (*P* < 0.01), and this trend further exacerbated by ESAT-6:c-di-AMP inoculation (*P* < 0.001) (**Figure 4B**).

### ESAT-6:c-di-AMP Inhibited the Differentiation of Macrophages in Lung

It has been identified that ESAT-6 is a key mycobacterial effector induced metabolic perturbations to drive the differentiation of macrophage into lipid loaded foamy macrophage (Singh et al., 2015). We found that both ESAT-6 alone and c-di-AMP alone markedly reduced the proportions of lung macrophages than the Naïve group (*P* < 0.05) (**Figure 4C** **)**, and ESAT-6:c-di-AMP resulted in an 11.2% reduction in macrophages than Naïve mice (*P* < 0.01) (**Figure 4C** **).** Neutrophils, belong to phagocytes as macrophages, are the most abundant cell type in the bronchoalveolar lavage of the active pulmonary TB patients (Liu et al., 2017). However, ESAT-6:c-di-AMP had no effect on the proportion of neutrophils, so did vaccination with ESAT-6 alone and c-di-AMP alone (**Figure 4C**).

### ESAT-6:c-di-AMP Induced the Differentiation of Lung ILCs

Innate lymphoid cells (ILCs) are located at mucosal site that respond quickly to invading pathogens (Liu et al., 2017; Gupta et al., 2018; Ardain et al., 2019). ILCs share features of both the innate and adaptive immune systems, and are categorized into three main subsets, ILC1, ILC2, and ILC3 (Geremia and Arancibia-Carcamo, 2017; Steigler et al., 2018). NK cells are included in ILC1 group now, and close to killer T cells, while other types of ILC executes similarly to helper T cells. Lung cells were stained for FCM and gated the cells referred to a previous reported study (Steigler et al., 2018) as shown in **Figure S2**. At 4 weeks after intranasal immunization, ESAT-6 alone had no effect on NK and ILC1, but inhibited ILC2 and ILC3 numbers (**Figures 4D–K**). However, ESAT-6:c-di-AMP induced significant increase of NK cells and ILC1 subset compared with ESAT-6 alone group (*P* < 0.05) (**Figures 4D, E, H, I** **)**, which strongly inferred the synergy between ESAT-6 and c-di-AMP. In subunit vaccine group, ILC2 and ILC3 numbers elevated 1.9-fold and 2.6-fold than the group vaccinated with ESAT-6 respectively, which was mainly due to c-di-AMP (**Figures 4F, G, J, K** **)**. Thus, ESAT-6:c-di-AMP induced the differentiations of ILCs in lung and may enhance the immune responses against Mtb infection.

### ESAT-6 and c-di-AMP Regulated Autophagy of Macrophages in Different Stages

**Figure 5:**
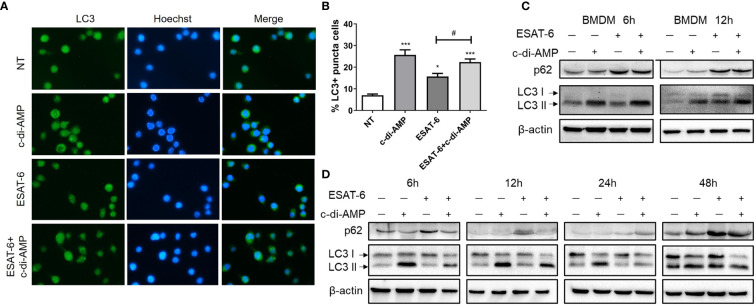
ESAT-6 and c-di-AMP regulated autophagy activation in macrophage. **(A)** Detection of LC3 puncta in RAW264.7 cells after cells were treated with c-di-AMP (10 μg/ml), ESAT-6 (10 μg/ml), and ESAT-6 co-administrated with c-di-AMP for 12 h by immunofluorescence staining. **(B)** Quantitative analysis of the cell proportion of LC3 puncta positive cells in **(A).** LC3 and p62/SQSTM expression in BMDMs **(C)** and MH-S cells **(D)** after cells were treated with c-di-AMP (10 μg/ml), ESAT-6 (10 μg/ml), or ESAT-6 co-stimulated with c-di-AMP at indicated time points. “*,” compared with the non-treatment group (NT) */^#^P<0.05, ***P<0.001.

Autophagy is increasingly appreciated as a pivotal mechanism by which macrophages defense intracellular bacteria including Mtb (Racanelli et al., 2018; Chai et al., 2019). It was reported that ESAT-6 inhibited autophagic flux by impeding autophagosome-lysosome fusion which involved in Mtb immune escape from macrophages (Dong et al., 2016; Peng and Sun, 2016; Wong, 2017). However, recombinant Mtb (rMtb) secreting more c-di-AMP could induce higher autophagy compared with wildtype, which resulted in attenuation of the intracellular growth of rMtb in macrophages J774.1 (Dey et al., 2015). By immunofluorescence staining, we detected an increasing of LC3 puncta formation induced by c-di-AMP, and a mild rise induced by ESAT-6 after 24 h treatment (**Figures 5A, B** **)**. We found that in BMDMs and MH-S cells, ESAT-6 blocked autophagic flux by inhibiting p62 degradation, and c-di-AMP obviously initiated autophagy with an increasing of symbolic autophagy LC3 II after 6 h treatment (**Figures 5C, D** **)**. LC3 II formation induced by c-di-AMP was smoothed by increasing of p62 degradation induced by ESAT-6 after 24 h treatment (**Figures 5C, D** **)**. In ESAT-6 plus c-di-AMP treated cells, autophagy was inhibited with p62 accumulation after 48 h treatment (**Figure 5D**). These observations suggested that ESAT-6 strongly induced the inhibition of p62 degradation at early stage of treatment, which seemed to stock the excessive autophagy of LC3 II formation co-induced by ESAT-6 and c-di-AMP as treatment time prolonged.

### ESAT-6 Combined With c-di-AMP Promoted Inflammatory Cytokine Releases in MH-S Cells

**Figure 6:**
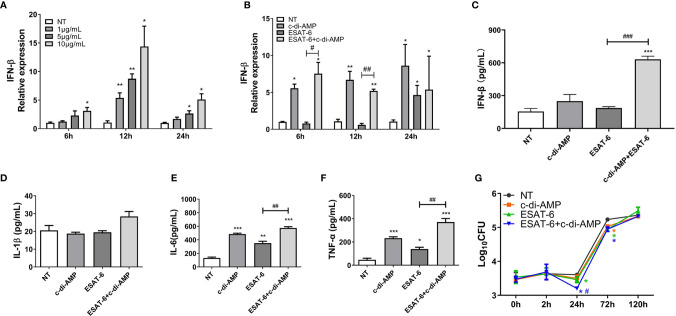
ESAT-6 combined with c-di-AMP promoted cytokine responses and inhibited Mtb survival in macrophages in early stage. **(A)** IFN-β mRNA levels of MH-S cells stimulated by c-di-AMP at different concentrations for indicated time period. **(B)** IFN-β mRNA levels of MH-S cells stimulated by c-di-AMP (10 μg/ml), ESAT-6 (10 μg/ml), or ESAT-6 co-stimulated with c-di-AMP for indicated time period. Cytokine secretions of IFN-β **(C)**, IL-β **(D)**, IL-6 **(E)**, and TNF-α **(F)** in the supernatants of MH-S cells stimulated by c-di-AMP (10 μg/ml), ESAT-6 (10 μg/ml), or ESAT-6 co-stimulated with c-di-AMP for 24 h. **(G)** Mtb H37Ra CFUs within MH-S cells post treatment with c-di-AMP (10 μg/ml), ESAT-6 (10 μg/ml), or ESAT-6 co-administrated with c-di-AMP for 24 h. Bacteria CFUs were determined at indicated time points. “*,” compared with the non-treatment group (NT); “#,” comparison between experimental groups as indicated in panels **(A–F)**. In panel **(G)**, “#” stands for comparison between ESAT-6+c-di-AMP and ESAT-6 treatments. */^#^P<0.05, **/^##^P<0.01, ***/^###^P<0.001.

c-di-AMP derived from mycobacteria triggers a type I IFN responses *via* the STING-TBK1-IRF3 axis in macrophages, which facilitates host resistance and clears intracellular bacterial infections (Yang et al., 2014; Dey et al., 2015). In our study, c-di-AMP induced IFN-β transcription after 6 h treatment at a dose-dependent manner and lasted at least 24 h (**Figure 6A**). It was reported that ESAT-6 induced IFN-β response *via* TLRs-mediated signaling in BMDMs and MH-S cells (Jang et al., 2018). We found that ESAT-6 induced IFN-β transcription (**Figure 6B** **)**, but not secretion after 24 h treatment (**Figure 6C**). As a result, ESAT-6 with c-di-AMP induced significant IFN-β secretion after 24 h treatment in MH-S cells which suggested that c-di-AMP had additive effect on ESAT-6 induced IFN-β response (**Figure 6C**). Neither ESAT-6 nor c-di-AMP stimulated IL-1β secretion in MH-S cells after 24 h treatment (**Figure 6D**), similarly to that of in lung and spleen of mice (**Figures 2H**, **3E**). Additionally, co-stimulation with ESAT-6 and c-di-AMP triggered elevated IL-6 and TNF-α secretions than ESAT-6 alone (**Figures 6E, F** **)**, consistent with the results obtained in mice (**Figures 2J, K** **)**. Stimulation of c-di-AMP or ESAT-6 alone induced significant IL-6 and TNF-α secretion (**Figures 6E, F** **)**.

### c-di-AMP Enhanced the Restriction of Mtb Survival Induced by ESAT-6 at Early Infection Stage

We wondered whether ESAT-6 and c-di-AMP treatment affects the intracellular growth of Mtb in MH-S cells. After 24 h treatment, ESAT-6 could significantly inhibit Mtb survivals in MH-S cells by 0.15 Log_10_CFU reduction (**Figure 6G** **)**. c-di-AMP enhanced the inhibition of ESAT-6 on Mtb survivals by 0.26 Log_10_CFU reduction, and the difference was significant between ESAT-6 and combined treatment, suggesting a synergy of ESAT-6 and c-di-AMP (**Figure 6G** **).** What is noticeable is that Mtb survivals significantly increased after 24 h of infection, and the inhibition effect of all treatment groups were vanished after 120 h of infection (**Figure 6G** **)**. It suggested that the rapidly activated innate immune response in macrophages could effectively resist early infection, while it was not enough for sustained Mtb infection. Thus, the activated adaptive immune response is needed to clear the infection finally.

### ESAT-6 Specific Antibody Declined in Immunized Mice After Mtb Infection

**Figure 7:**
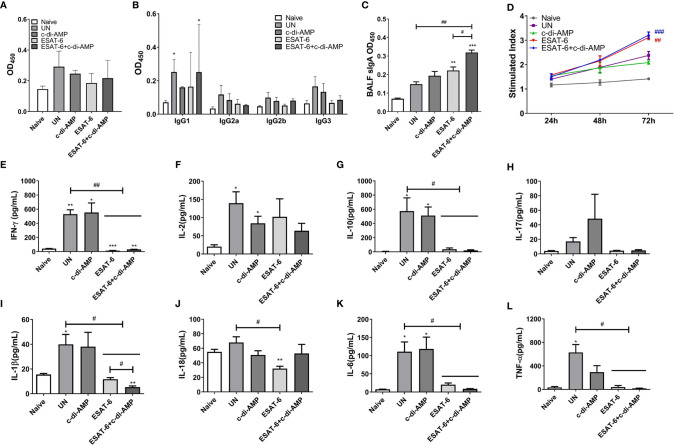
Subunit vaccine ESAT-6:c-di-AMP elicited mucosal sIgA *in vivo* and resulted in restricted cellular immune response *in vitro* after Mtb infection. ESAT-6 specific IgG **(A)** and antibody subclass **(B)** in sera (1:200) of immunized mice after Mtb infection. **(C)** ESAT-6 specific sIgA in BALF of immunized mice after Mtb infection. **(D)** Splenocytes proliferation after cells stimulated with ESAT-6 (5 μg/ml) at indicated time points *in vitro*. **(E–L)** Splenocytes stimulated by ESAT-6 (5 μg/ml) for 72 h *in vitro* and supernatants were examined for cytokines using ELISA. “*,” compared with the control group (Naïve); “#,” comparison between experimental groups as indicated in panels **(A, C, E–L)**. In panel **(D)**, “#” stands for compared with UN group. */^#^P<0.05, **/^##^P<0.01, ***/^###^P<0.001.

After Mtb infection, ESAT-6 specific IgG level of sera declined in immunized mice (**Figure 7A**), and IgG subclasses were almost undetectable in most groups, excepted IgG1 (**Figure 7B**). Mucosal local humoral response of specific sIgA levels were higher in ESAT-6 alone immunized mice in BALF compared with the un-immunized group after Mtb infection (*P* < 0.05) (**Figure 7C** **)**. Still, ESAT-6:c-di-AMP immunized mice exhibited higher sIgA levels than those of ESAT-6 group (*P* < 0.05) (**Figure 7C**), which showed similar trend as that of after immunization (**Figure 1D**).

### Splenocytes of ESAT-6 Immunized Mice Exhibited Anergic Cytokine Response After Mtb Infection

It was obviously that ESAT-6 induced splenocytes proliferation after Mtb infection (*P* < 0.05), and the proliferation stimulated by c-di-AMP faded away after Mtb infection (**Figure 7D**). Splenocytes from Mtb infection mice without vaccination produced significant Th1/Th2 and inflammatory cytokines after re-stimulated with ESAT-6 (**Figures 7E–L** **)**. To our surprise, splenocytes from ESAT-6 and ESAT-6:c-di-AMP immunization groups were not responsive to the re-stimulation of ESAT-6 *in vitro* (**Figures 7E–L**). Almost all cytokine levels detected, except for IL-2, were similarly to that of Naïve mice in supernatants of splenocytes from ESAT-6 and ESAT-6:c-di-AMP immunization mice. At the meantime, c-di-AMP did not affect the productions of cytokines after Mtb infection (**Figures 7E–L** **)**. Our study showed that splenocytes were initially activated by ESAT-6:c-di-AMP immunization in mice (**Figures 2D–K** **)**, and then re-activated by Mtb infection *in vivo*. After Mtb challenge, splenocytes showed not responsive to antigen re-stimulation *in vitro*, concluded with low levels of cytokine releases. Besides, c-di-AMP as adjuvant exacerbated the inhibitory of splenocytes on IL-1β release (**Figure 7I**).

### ESAT-6:c-di-AMP Conferred Protection Against Mtb Infection by Vein

**Figure 8:**
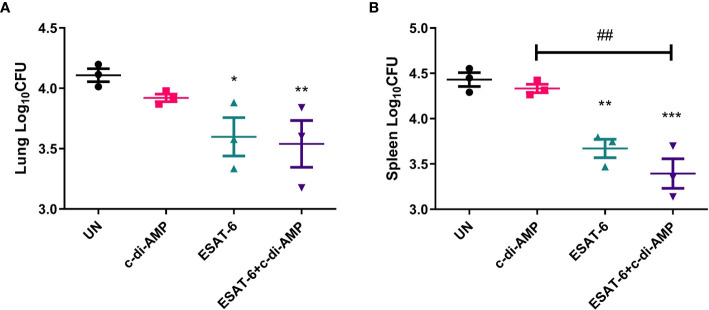
Bacterial burdens in mice after Mtb challenge. After 12-week of Mtb infection, bacterial burdens in the lungs **(A)** and spleens **(B)** of un-immunized (UN), c-di-AMP or ESAT-6 alone, and ESAT-6:c-di-AMP, respectively, were counted by plating. “*,” compared with un-immunized group (UN) *P<0.05, **/^##^P<0.01, ***P<0.001.

In lung, vaccination of ESAT-6:c-di-AMP reduced bacterial load by 0.57 Log_10_CFU than UN (*P* < 0.01) (**Figure 8A**). In spleen, ESAT-6:c-di-AMP vaccination group showed 1.06 Log_10_CFU (*P* < 0.001) reduction in bacterial load than the UN group (**Figure 8B** **)**. And ESAT-6 vaccination alone exhibited 0.78 Log_10_CFU (*P* < 0.01) reduction in bacterial load than UN mice (**Figure 8B**). Overall, ESAT-6 immunization by mucosal route could reduce Mtb CFUs in lung (*P* < 0.05) and in spleen (*P* < 0.01) (**Figures 8A, B** **)**. While, c-di-AMP immunization alone could not provide significant protection against Mtb infection intravenously (**Figure 8A** **)**, which was similar with our results from rBCG with elevated c-di-AMP (Ning et al., 2019). Though no differences were found between subunit vaccine ESAT-6:c-di-AMP and ESAT-6 alone immunization, there exhibited a further downward trend in ESAT-6:c-di-AMP immunized group. These results proved that subunit vaccine ESAT-6:c-di-AMP inoculation by mucosal route might provide protection against Mtb infection intravenously, which was from synergistic effects of ESAT-6 and c-di-AMP.

## Discussion

Several prophylactic subunit vaccines for TB have been tested in multiple animal models of mouse, guinea pig, and non-human primate. ESAT-6 is one of the most promising candidates for TB vaccine design. Previously, we have demonstrated that Ag85B-ESAT-6 adjuvanted with MPLA induces significant humoral and cellular immune response *via* s.c. administration, which mainly caused by Ag85B (Xu, 2014). We found that levels of anti-ESAT-6 antibody were increased, but very low in infected animals, as well as in patients (**Figure S1**), and adjuvant enhanced immunogenicity of ESAT-6 either *via* s.c. or i.m. route (Lu et al., 2018). A vaccine formulated with ESAT-6 adjuvanted with both aluminum hydroxide and TLR8 agonist immunized by i.m. route provided protection against Mtb challenge in vein (Tang et al., 2017). Subunit vaccine based on Ag85B-Acr-ESAT6-HBHA intranasal boost administration provided protection against Mtb infection in BCG-primed mice (Hart et al., 2018). Moreover, a mucosal vaccine based on ESAT6_1-20_ peptide delivered through the mucosal route inducing IL-17-dependent induction of CXCL13 and provide protection against Mtb infection in mice (Gopal et al., 2013). These studies show that ESAT-6 as component of subunit vaccine could provide protection against Mtb infection, but requires adjuvant or vaccination through mucosal route to enhance its effects.

Recent study reported that Ag85B-ESAT-6 antigen, delivered by immunogenic carrier of *Lactobacillus plantarum* adjuvanted with poly(I:C) through a primary subcutaneous immunization followed by intranasal boosters, led to slightly elevated IgG levels in serum, and significantly increased levels of antigen-specific mucosal IgA in mice (Kuczkowska et al., 2019). In this study, we composed of a subunit vaccine ESAT-6:c-di-AMP for mucosal immunization. Though IgG were not elevated significantly by addition of c-di-AMP (**Figure 1B**), our study showed that subunit vaccine ESAT-6:c-di-AMP administrated by intranasal route induced higher mucosal sIgA in BALF (**Figure 1C**), which means protection against respiratory pathogens.

ESAT-6 is potent antigen for human T cells and is a putative vaccine candidate against Mtb infection. In this study, ESAT-6:c-di-AMP dramatically improved the production of Th1/Th2/Th17 as well as inflammatory cytokines responses by mucosal inoculation (**Figures 2D–K**), which is in line of previous study of c-di-AMP as mucosal adjuvant (Ebensen et al., 2011). However, it seemed that the profile of cytokine responses was different between lung (**Figure 3**) and spleen (**Figures 2D–K**). From these results, we proposed that ESAT-6 and c-di-AMP may have direct effects on immune cells at mucosal site. CD4^+^ T cells is more sensitive to recognize Mtb-infected macrophages than CD8^+^ T cells in lungs, which could correlate with protective immunity (Patankar et al., 2020). The proportions of cell subsets in mice lungs showed that ESAT-6 inhibited CD8^+^ T cells, and c-di-AMP exacerbate the decline of CD8^+^ T cells induced by ESAT-6 in ESAT-6:c-di-AMP group (**Figure 4B**). CD8^+^ T cells, also named cytotoxic T lymphocytes (CTL), killing mechanism is generally dependent on the production of perforin (Lin and Flynn, 2015). While, perforin knockout mice were not more susceptible to Mtb infection (Cooper et al., 1997), but had higher overall IFN-γ production (Serbina et al., 2001), which could compensate for the lack of CD8^+^ T cell cytotoxicity. As a result, we found that ESAT-6:c-di-AMP inoculation triggered enhanced overall IFN-γ response. Animal model data support a non-redundant role for CD8^+^ T cells in control of Mtb infection (Lin and Flynn, 2015). Besides, ESAT-6:c-di-AMP induced significant increase of NK cells, which belong to ILC1 subsets, a group of quick response immune cells at mucosal site. Thus, ESAT-6 and c-di-AMP may stimulate immune cells through the same or different mechanisms.

The mechanism study with murine alveolar macrophage cell line MH-S also found the divergence of ESAT-6 and c-di-AMP on immune responses. ESAT-6 alone unable to induce IFN-β production, which was inconsistent with report of ESAT-6 alone promoted IFN-β mRNA level in MH-S cells (Jang et al., 2018). Co-stimulation with ESAT-6 and c-di-AMP promoted IFN-β response, which was mainly due to c-di-AMP (**Figure 3I**). Autophagy is an important innate immune mechanism against intracellular bacteria such as Mtb. ESAT-6 inhibited autophagic flux as reported before (Dong et al., 2016; Peng and Sun, 2016; Wong, 2017). However, c-di-AMP induced increasing of autophagy-associated genes expression in RAW264.7 (Ning et al., 2019), and LC3 II formation in J774.1 (Dey et al., 2015) with elevated c-di-AMP in mycobacteria. In this study, we found that c-di-AMP could triggered LC3 II formation at early stage, but enhanced the p62 degradation inhibition by ESAT-6 and finally inhibited autophagy with the decrease of LC3 II formation after long-term stimulation (**Figures 5C, D** **)** with more IL-1β, IL-6, and TNF-α secretions (**Figures 6D–F**). The survival trends were similarly to that of autophagy activated with either ESAT-6 or c-di-AMP alone or combined, consistent with the consensus that autophagy plays a major role in restricting bacterial replication (Paik et al., 2019).

After Mtb infection, ESAT-6 specific IgG levels declined in immunized groups compared to those of Naïve mice (**Figures 7A, B**), but high sIgA maintained in ESAT-6 alone and ESAT-6:c-di-AMP immunized mice (**Figure 7C** **)**. Though ESAT-6 induced splenocytes proliferation after Mtb infection, ESAT-6 immunized mice showed no response to antigen re-stimulation and almost no longer secreted cytokines, including Th1/Th2/Th17 as well as inflammatory cytokines *in vitro* (**Figures 7E–H**). It was reported that ESAT-6 treated human T cells, pre-activated with anti-CD3/CD28 mAbs or heat-killed Mtb, produced less IFN-γ compared with non-treatment (Wang et al., 2009). It was also revealed that ESAT-6 can directly bind to T cells and subsequently inhibits the production of IFN-γ by activated T cells through p38 mitogen-activated protein kinase (MAKP)-dependent pathway (Wang et al., 2009). ESAT-6 also inhibited the production of IL-10, IL-17, and TNF-α, but did not affect IL-2 production (Wang et al., 2009; Peng et al., 2011). Besides, ESAT-6 primes DC to stimulate Th17 and inhibits Th1 immune responses, and effects of ESAT-6 were not mediated through cAMP or p38 MAPK pathway (Wang et al., 2012). These observations may partly explain the unresponsive state of splenocytes from ESAT-6:c-di-AMP immunized mice after Mtb infection.

For the evaluation of protection efficiency, we chose Mtb H37Ra strain, a risk group (RG2) pathogen of H37Rv relevant, which has also been extensive used as a surrogate to study the virulence of Mtb in Biosafety Level 2 (BSL2) facilities (Ning et al., 2017; Yang et al., 2020). Mucosal vaccination can induce local mucosal and systemic immune responses. Both ESAT-6 and ESAT-6:c-di-AMP subunit vaccines provided protection against intravenous Mtb H37Ra infection by intranasally in mice. C-di-AMP as adjuvant could improve the protection efficiency of ESAT-6 to a certain extent, while no significant differences were found between two ESAT-6 groups (**Figure 8A**). Our previous study showed that rBCG with c-di-AMP as adjuvant could induce higher immune responses, but provided similar protection as BCG did against Mtb infection intravenously (Ning et al., 2019). However, a similar rBCG conferred improved protection than BCG after respiratory infection of Mtb in guinea pig (Dey et al., 2020). Another study suggested that c-di-AMP as a relatively new immunomodulatory molecule exhibits great potential to promote protective immunity and as an immune-adjuvant to enhance vaccine potency (Libanova et al., 2012). Additionally, another inoculation route of s.c. were applied which c-di-AMP exhibited superior properties in targeting DC (Volckmar et al., 2019). Based on multiple functions that c-di-AMP involved, we will further evaluate the immune protection of these subunit vaccine through more susceptible animals, such as guinea pigs, aerosol challenge of Mtb, or different immunization pathways and strategies.

In this study, there were no adverse reactions observed in the mice throughout the experiments. Of note, ESAT-6 is considered as a virulence factor of Mtb since its knock-out strain showed attenuated virulence in animal infection models. And c-di-AMP promoted a self-limited immune activation by targeting STING degradation, which is a prerequisite for designing the vaccines with predictable efficacy and safety profiles (Rueckert et al., 2017). Nevertheless, subunit vaccine delivered by mucosal route needs a more careful assessment of their safety profile and their capacity to promote potential adverse effects. In this study, we demonstrated that intranasal inoculation of a subunit vaccine ESAT-6:c-di-AMP promoted humoral and cellular immune response, which provided preliminary evidences that antigen adjuvanted with c-di-AMP might be used to formulate mucosal vaccines against Mtb infection.

## Data Availability Statement

The original contributions presented in the study are included in the article/**Supplementary Material**. Further inquiries can be directed to the corresponding authors.

## Ethics Statement

The animal studies were conducted under the approval of the Institutional Ethics Committee of Tangdu Hospital, Second Affiliated Hospital of Air Force Medical University, according to the recommendations from the Guide for the Care and Use of Laboratory Animals of the Institute (Approval No. TDLL-2016325).

## Author Contributions

HN, WZ, and JK performed most experiments. XL, YL, CG, WS, and HW conducted several experiments. HN, WZ, and TD analyzed the data. HN and YB wrote the manuscript. YB and LS conceived and designed the research. YB supervised this work. All authors contributed to the article and approved the submitted version.

## Funding

This study was funded by National Major Special Projects of 13th Five-year Plan (No. 2018ZX10302302002004), National Natural Science Foundation (No. 81671638, 81971560), Provincial Natural Science Foundation of Shaanxi Province (No. 2018ZDXM-SF-004).

## Conflict of Interest

The authors declare that the research was conducted in the absence of any commercial or financial relationships that could be construed as a potential conflict of interest.

## References

[B1] Abdallah A. M. Gey van Pittius N. C. Champion P. A. Cox J. Luirink J. Vandenbroucke-Grauls C. M.. (2007). Type VII secretion–mycobacteria show the way. Nat. Rev. Microbiol. 5, 883–891. 10.1038/nrmicro1773 17922044

[B2] Ardain A. Domingo-Gonzalez R. Das S. Kazer S. W. Howard N. C. Singh A.. (2019). Group 3 innate lymphoid cells mediate early protective immunity against tuberculosis. Nature 570, 528–532. 10.1038/s41586-019-1276-2 31168092 PMC6626542

[B3] Burdette D. L. Monroe K. M. Sotelo-Troha K. Iwig J. S. Eckert B. Hyodo M.. (2011). STING is a direct innate immune sensor of cyclic di-GMP. Nature 478, 515–518. 10.1038/nature10429 21947006 PMC3203314

[B4] Chai Q. Wang X. Qiang L. Zhang Y. Ge P. Lu Z.. (2019). A *Mycobacterium tuberculosis* surface protein recruits ubiquitin to trigger host xenophagy. Nat. Commun. 10, 1973. 10.1038/s41467-019-09955-8 31036822 PMC6488588

[B5] Commichau F. M. Heidemann J. L. Ficner R. Stulke J. (2019). Making and Breaking of an Essential Poison: the Cyclases and Phosphodiesterases That Produce and Degrade the Essential Second Messenger Cyclic di-AMP in Bacteria. J. Bacteriol. 201, e00462–18. 10.1128/JB.00462-18 PMC6287462 30224435

[B6] Cooper A. M. D’Souza C. Frank A. A. Orme I. M. (1997). The course of *Mycobacterium tuberculosis* infection in the lungs of mice lacking expression of either perforin- or granzyme-mediated cytolytic mechanisms. Infect. Immun. 65, 1317–1320. 10.1128/IAI.65.4.1317-1320.1997 9119468 PMC175134

[B7] Copland A. Diogo G. R. Hart P. Harris S. Tran A. C. Paul M. J.. (2018). Mucosal Delivery of Fusion Proteins with *Bacillus subtilis* Spores Enhances Protection against Tuberculosis by Bacillus Calmette-Guerin. Front. Immunol. 9:346:346. 10.3389/fimmu.2018.00346 29593708 PMC5857916

[B8] Devaux L. Kaminski P. A. Trieu-Cuot P. Firon A. (2018). Cyclic di-AMP in host-pathogen interactions. Curr. Opin. Microbiol. 41, 21–28. 10.1016/j.mib.2017.11.007 29169058

[B9] Dey B. Dey R. J. Cheung L. S. Pokkali S. Guo H. Lee J. H.. (2015). A bacterial cyclic dinucleotide activates the cytosolic surveillance pathway and mediates innate resistance to tuberculosis. Nat. Med. 21, 401–406. 10.1038/nm.3813 25730264 PMC4390473

[B10] Dey R. J. Dey B. Singh A. K. Praharaj M. Bishai W. (2020). Bacillus Calmette-Guerin Overexpressing an Endogenous Stimulator of Interferon Genes Agonist Provides Enhanced Protection Against Pulmonary Tuberculosis. J. Infect. Dis. 221, 1048–1056. 10.1093/infdis/jiz116 30901058 PMC7931846

[B11] Dong H. Jing W. Runpeng Z. Xuewei X. Min M. Ru C.. (2016). ESAT6 inhibits autophagy flux and promotes BCG proliferation through MTOR. Biochem. Biophys. Res. Commun. 477, 195–201. 10.1016/j.bbrc.2016.06.042 27317487

[B12] Ebensen T. Libanova R. Schulze K. Yevsa T. Morr M. Guzman C. A. (2011). Bis-(3’,5’)-cyclic dimeric adenosine monophosphate: strong Th1/Th2/Th17 promoting mucosal adjuvant. Vaccine 29, 5210–5220. 10.1016/j.vaccine.2011.05.026 21619907

[B13] Geremia A. Arancibia-Carcamo C. V. (2017). Innate Lymphoid Cells in Intestinal Inflammation. Front. Immunol. 8:1296:1296. 10.3389/fimmu.2017.01296 29081776 PMC5645495

[B14] Gopal R. Rangel-Moreno J. Slight S. Lin Y. Nawar H. F. Fallert Junecko B. A.. (2013). Interleukin-17-dependent CXCL13 mediates mucosal vaccine-induced immunity against tuberculosis. Mucosal Immunol. 6, 972–984. 10.1038/mi.2012.135 23299616 PMC3732523

[B15] Gupta N. Kumar R. Agrawal B. (2018). New Players in Immunity to Tuberculosis: The Host Microbiome, Lung Epithelium, and Innate Immune Cells. Front. Immunol. 9:709:709. 10.3389/fimmu.2018.00709 29692778 PMC5902499

[B16] Hart P. Copland A. Diogo G. R. Harris S. Spallek R. Oehlmann W.. (2018). Nanoparticle-Fusion Protein Complexes Protect against *Mycobacterium tuberculosis* Infection. Mol. Ther. 26, 822–833. 10.1016/j.ymthe.2017.12.016 29518353 PMC5910664

[B17] Jang A. R. Choi J. H. Shin S. J. Park J. H. (2018). *Mycobacterium tuberculosis* ESAT6 induces IFN-beta gene expression in Macrophages via TLRs-mediated signaling. Cytokine 104, 104–109. 10.1016/j.cyto.2017.10.006 29046251

[B18] Jung B. G. Wang X. Yi N. Ma J. Turner J. Samten B. (2017). Early Secreted Antigenic Target of 6-kDa of *Mycobacterium tuberculosis* Stimulates IL-6 Production by Macrophages through Activation of STAT3. Sci. Rep. 7:40984. 10.1038/srep40984 28106119 PMC5247711

[B19] Kuczkowska K. Overland L. Rocha S. D. C. Eijsink V. G. H. Mathiesen G. (2019). Comparison of eight *Lactobacillus* species for delivery of surface-displayed mycobacterial antigen. Vaccine 37, 6371–6379. 10.1016/j.vaccine.2019.09.012 31526620

[B20] Libanova R. Becker P. D. Guzman C. A. (2012). Cyclic di-nucleotides: new era for small molecules as adjuvants. Microbial. Biotechnol. 5, 168–176. 10.1111/j.1751-7915.2011.00306.x PMC3815777 21958423

[B21] Lin P. L. Flynn J. L. (2015). CD8 T cells and *Mycobacterium tuberculosis* infection. Semin. Immunopathol. 37, 239–249. 10.1007/s00281-015-0490-8 25917388 PMC4439333

[B22] Liu C. H. Liu H. Ge B. (2017). Innate immunity in tuberculosis: host defense vs pathogen evasion. Cell Mol. Immunol. 14, 963–975. 10.1038/cmi.2017.88 28890547 PMC5719146

[B23] Lu Y. Kang J. Ning H. Wang L. Xu Y. Xue Y.. (2018). Immunological characteristics of *Mycobacterium tuberculosis* subunit vaccines immunized through different routes. Microb. Pathog. 125, 84–92. 10.1016/j.micpath.2018.09.009 30195646

[B24] Lycke N. (2012). Recent progress in mucosal vaccine development: potential and limitations. Nat. Rev. Immunol. 12, 592–605. 10.1038/nri3251 22828912

[B25] Macpherson A. J. McCoy K. D. Johansen F. E. Brandtzaeg P. (2008). The immune geography of IgA induction and function. Mucosal Immunol. 1, 11–22. 10.1038/mi.2007.6 19079156

[B26] Mangtani P. Abubakar I. Ariti C. Beynon R. Pimpin L. Fine P. E.. (2014). Protection by BCG vaccine against tuberculosis: a systematic review of randomized controlled trials. Clin. Infect. Dis. Off. Publ. Infect. Dis. Soc. America 58, 470–480. 10.1093/cid/cit790 24336911

[B27] Mantovani A. Dinarello C. A. Molgora M. Garlanda C. (2019). Interleukin-1 and Related Cytokines in the Regulation of Inflammation and Immunity. Immunity 50, 778–795. 10.1016/j.immuni.2019.03.012 30995499 PMC7174020

[B28] Matos M. N. Cazorla S. I. Schulze K. Ebensen T. Guzman C. A. Malchiodi E. L. (2017). Immunization with Tc52 or its amino terminal domain adjuvanted with c-di-AMP induces Th17+Th1 specific immune responses and confers protection against *Trypanosoma cruzi* . PloS Negl. Trop. Dis. 11, e0005300. 10.1371/journal.pntd.0005300 28234897 PMC5342303

[B29] Namvarpour M. Tebianian M. Mansouri R. Ebrahimi S. M. Kashkooli S. (2019). Comparison of different immunization routes on the immune responses induced by *Mycobacterium tuberculosis* ESAT-6/CFP-10 recombinant protein. Biologicals 59, 6–11. 10.1016/j.biologicals.2019.04.002 31014910

[B30] Ning H. Lu Y. Kang J. Xu Y. Wang L. Wang Y.. (2017). Establishment of mouse models of persistent tuberculosis and characteristics of that infection. J. Pathogen. Biol. 12, 219–223. 10.13350/j.cjpb.170306

[B31] Ning H. Wang L. Zhou J. Lu Y. Kang J. Ding T.. (2019). Recombinant BCG With Bacterial Signaling Molecule Cyclic di-AMP as Endogenous Adjuvant Induces Elevated Immune Responses After *Mycobacterium tuberculosis* Infection. Front. Immunol. 10:1519:1519. 10.3389/fimmu.2019.01519 31333655 PMC6618344

[B32] Paik S. Kim J. K. Chung C. Jo E. K. (2019). Autophagy: A new strategy for host-directed therapy of tuberculosis. Virulence 10, 448–459. 10.1080/21505594.2018.1536598 30322337 PMC6550549

[B33] Paquin-Proulx D. Costa P. R. Terrassani Silveira C. G. Marmorato M. P. Cerqueira N. B. Sutton M. S.. (2018). Latent *Mycobacterium tuberculosis* Infection Is Associated With a Higher Frequency of Mucosal-Associated Invariant T and Invariant Natural Killer T Cells. Front. Immunol. 9:1394:1394. 10.3389/fimmu.2018.01394 29971068 PMC6018487

[B34] Patankar Y. R. Sutiwisesak R. Boyce S. Lai R. Lindestam Arlehamn C. S. Sette A.. (2020). Limited recognition of *Mycobacterium tuberculosis*-infected macrophages by polyclonal CD4 and CD8 T cells from the lungs of infected mice. Mucosal Immunol. 13, 140–148. 10.1038/s41385-019-0217-6 31636345 PMC7161428

[B35] Peng X. Sun J. (2016). Mechanism of ESAT-6 membrane interaction and its roles in pathogenesis of *Mycobacterium tuberculosis* . Toxicon. 116, 29–34. 10.1016/j.toxicon.2015.10.003 26456678 PMC4973572

[B36] Peng H. Wang X. Barnes P. F. Tang H. Townsend J. C. Samten B. (2011). The *Mycobacterium tuberculosis* early secreted antigenic target of 6 kDa inhibits T cell interferon-gamma production through the p38 mitogen-activated protein kinase pathway. J. Biol. Chem. 286, 24508–24518. 10.1074/jbc.M111.234062 21586573 PMC3129230

[B37] Racanelli A. C. Kikkers S. A. Choi A. M. K. Cloonan S. M. (2018). Autophagy and inflammation in chronic respiratory disease. Autophagy 14, 221–232. 10.1080/15548627.2017.1389823 29130366 PMC5902194

[B38] Rueckert C. Rand U. Roy U. Kasmapour B. Strowig T. Guzman C. A. (2017). Cyclic dinucleotides modulate induced type I IFN responses in innate immune cells by degradation of STING. FASEB J. Off. Publ. Fed. Am. Soc. Exp. Biol. 31, 3107–3115. 10.1096/fj.201601093R 28396343

[B39] Sanchez M. V. Ebensen T. Schulze K. Cargnelutti D. Blazejewska P. Scodeller E. A.. (2014). Intranasal delivery of influenza rNP adjuvanted with c-di-AMP induces strong humoral and cellular immune responses and provides protection against virus challenge. PloS One 9, e104824. 10.1371/journal.pone.0104824 25140692 PMC4139298

[B40] Serbina N. V. Lazarevic V. Flynn J. L. (2001). CD4(+) T cells are required for the development of cytotoxic CD8(+) T cells during *Mycobacterium tuberculosis* infection. J. Immunol. 167, 6991–7000. 10.4049/jimmunol.167.12.6991 11739519

[B41] Shaw D. M. Merien F. Braakhuis A. Dulson D. (2018). T-cells and their cytokine production: The anti-inflammatory and immunosuppressive effects of strenuous exercise. Cytokine 104, 136–142. 10.1016/j.cyto.2017.10.001 29021092

[B42] Singh V. Kaur C. Chaudhary V. K. Rao K. V. Chatterjee S. (2015). *M. tuberculosis* Secretory Protein ESAT-6 Induces Metabolic Flux Perturbations to Drive Foamy Macrophage Differentiation. Sci. Rep. 5:12906. 10.1038/srep12906 26250836 PMC5388048

[B43] Skrnjug I. Guzman C. A. Rueckert C. (2014). Cyclic GMP-AMP displays mucosal adjuvant activity in mice. PloS One 9, e110150. 10.1371/journal.pone.0110150 25295996 PMC4190368

[B44] Steigler P. Daniels N. J. McCulloch T. R. Ryder B. M. Sandford S. K. Kirman J. R. (2018). BCG vaccination drives accumulation and effector function of innate lymphoid cells in murine lungs. Immunol. Cell Biol. 96, 379–389. 10.1111/imcb.12007 29363172

[B45] Stylianou E. Paul M. J. Reljic R. McShane H. (2019). Mucosal delivery of tuberculosis vaccines: a review of current approaches and challenges. Expert Rev. Vaccines 18, 1271–1284. 10.1080/14760584.2019.1692657 31876199 PMC6961305

[B46] Tang J. Sun M. Shi G. Xu Y. Han Y. Li X.. (2017). Toll-Like Receptor 8 Agonist Strengthens the Protective Efficacy of ESAT-6 Immunization to *Mycobacterium tuberculosis* Infection. Front. Immunol. 8:1972:1972. 10.3389/fimmu.2017.01972 29416532 PMC5787779

[B47] Unnikrishnan M. Constantinidou C. Palmer T. Pallen M. J. (2017). The Enigmatic Esx Proteins: Looking Beyond Mycobacteria. Trends Microbiol. 25, 192–204. 10.1016/j.tim.2016.11.004 27894646

[B48] Volckmar J. Knop L. Stegemann-Koniszewski S. Schulze K. Ebensen T. Guzman C. A.. (2019). The STING activator c-di-AMP exerts superior adjuvant properties than the formulation poly(I:C)/CpG after subcutaneous vaccination with soluble protein antigen or DEC-205-mediated antigen targeting to dendritic cells. Vaccine 37, 4963–4974. 10.1016/j.vaccine.2019.07.019 31320219

[B49] Wang X. Barnes P. F. Dobos-Elder K. M. Townsend J. C. Chung Y. T. Shams H.. (2009). ESAT-6 inhibits production of IFN-gamma by *Mycobacterium tuberculosis*-responsive human T cells. J. Immunol. 182, 3668–3677. 10.4049/jimmunol.0803579 19265145 PMC5488288

[B50] Wang X. Barnes P. F. Huang F. Alvarez I. B. Neuenschwander P. F. Sherman D. R.. (2012). Early secreted antigenic target of 6-kDa protein of *Mycobacterium tuberculosis* primes dendritic cells to stimulate Th17 and inhibit Th1 immune responses. J. Immunol. 189, 3092–3103. 10.4049/jimmunol.1200573 22904313 PMC3436987

[B51] Wong K.-W. (2017). The Role of ESX-1 in *Mycobacterium tuberculosis* Pathogenesis. Microbiol. Spectrum. 5. 10.1128/microbiolspec.TBTB2-0001-2015 PMC11687508 28513416

[B52] Woodward J. J. Iavarone A. T. Portnoy D. A. (2010). c-di-AMP secreted by intracellular *Listeria monocytogenes* activates a host type I interferon response. Science 328, 1703–1705. 10.1126/science.1189801 20508090 PMC3156580

[B53] World Health Organization (2020). Global Tuberculosis Report. Available at: https://www.who.int/tb/publications/global_report/esn/ (Accessed March 5, 2020).

[B54] Xu C. (2014). Preliminary evaluation of a subunit vaccine based on fusion proteins for its therapeutic efficacy in Mycobacterium tuberculosis infected guinea pig and mouse modle. [dissertation/master’s thesis] ([Xi’an (Shaanxi)]: Air Force Medical University).

[B55] Yang J. Bai Y. Zhang Y. Gabrielle V. D. Jin L. Bai G. (2014). Deletion of the cyclic di-AMP phosphodiesterase gene (cnpB) in *Mycobacterium tuberculosis* leads to reduced virulence in a mouse model of infection. Mol. Microbiol. 93, 65–79. 10.1111/mmi.12641 24806618 PMC4088933

[B56] Yang S. J. Chen Y. Y. Hsu C. H. Hsu C. W. Chang C. Y. Chang J. R.. (2020). Activation of M1 Macrophages in Response to Recombinant TB Vaccines With Enhanced Antimycobacterial Activity. Front. Immunol. 11:1298:1298. 10.3389/fimmu.2020.01298 32655570 PMC7325470

[B57] Zarrella T. M. Bai G. (2020). The many roles of the bacterial second messenger cyclic di-AMP in adapting to stress cues. J. Bacteriol. 203, e00348–20. 10.1128/JB.00348-20 PMC7723955 32839175

[B58] Zhu B. Dockrell H. M. Ottenhoff T. H. M. Evans T. G. Zhang Y. (2018). Tuberculosis vaccines: Opportunities and challenges. Respirology 23, 359–368. 10.1111/resp.1324 29341430

