# Supplementary material for: Subunit Vaccine ESAT-6:c-di-AMP Delivered by Intranasal Route Elicits Immune Responses and Protects Against Mycobacterium tuberculosis Infection
Source: Front Cell Infect Microbiol. 2021 Mar 22;11:647220. doi: 10.3389/fcimb.2021.647220 (PMC8019782; doi:10.3389/fcimb.2021.647220)
Supplement: Supplementary file 1 [file DataSheet_1.pdf]

Table S1 Antibodies used for flow cytometry of this study

Marker/Function	Species	Fluorescein	Clone	Source
Zombie NIR™ Fixable Viability Kit	/	Zombie NIR	/	BioLegend
TruStain FcX™ PLUS (anti-mouse CD16/32)	Mouse	/	S17011E	BioLegend
CD3	Mouse	Brilliant Violet 510	17A2	BioLegend
CD3	Mouse	PerCP	17A2	BioLegend
CD4	Mouse	PE-Cy7	RM4-5	BioLegend
CD8	Mouse	PE	53-6.7	BioLegend
CD8	Mouse	Brilliant Violet 510	53-6.7	BioLegend
CD19	Mouse	Pacific Blue	6D5	BioLegend
CD49b	Mouse	PerCp/Cy5.5	DX5	BioLegend
CD11b	Mouse	APC	M1/70	BioLegend
Ly-6G	Mouse	FITC	1A8	BioLegend
IFN-γ	Mouse	FITC	XMG1.2	BioLegend
IL-2	Mouse	PE	JES6-5H4	BioLegend
IL-10	Mouse	APC	JES5-16E3	BioLegend
Lineage Cocktail with Isotype Ctrl	Mouse	Pacific Blue	CD3, 17A2; Ly-6G/Ly-6(C) RB6-8C5; CD11(B) M1/70; CD45R/B220, RA3-6B2; TER-119/Erythroid cells, Ter-119	BioLegend
IL-17RB	Mouse	APC	9B10	BioLegend
IL-23R	Mouse	PE	12B2B64	BioLegend
CD335 (NKp46)	Mouse	Brilliant Violet 605	29A1.4	BioLegend
CD90.2 (Thy1.2)	Mouse	PerCP	53-2.1	BioLegend
CD335 (NKp46)	Mouse	Brilliant Violet 605	29A1.4	BioLegend
CD127 (IL-7Rα)	Mouse	Alexa Fluor 488	A7R34	BioLegend

Table S2 Primers used in this study

Sequence (5' to 3')	Purpose
ACAACTTTGGCATTGTGGAA	qRT-PCR primer of Mus *gapdh* forward
GATGCAGGGATGATGTTCTG	qRT-PCR primer of Mus *gapdh* reverse
GCTCTGAGACAATGAACGCTAC	qRT-PCR primer of Mus *ifn-γ*, forward
TGCAGGATTTTCATGTCACC	qRT-PCR primer of Mus *ifn-γ*, reverse
CTTGTGCTCCTTGTCAACAG	qRT-PCR primer of Mus *il-2*, forward
TCCAAGTTCATCTTCTAGGC	qRT-PCR primer of Mus *il-2*, reverse
TGCTCTTACTGACTGGCAT	qRT-PCR primer of Mus *il-10*, forward
CTGGATCATTTCCGATAAGGC	qRT-PCR primer of Mus *il-10*, reverse
CCACGTCACCCTGGACTCTC	qRT-PCR primer of Mus *il-17a*, forward
CTCCGCATTGACACAGCG	qRT-PCR primer of Mus *il-17a*, reverse
AGCTCCAAGAAAGGACGAACA	qRT-PCR primer of Mus *ifn-β*, forward
GCCCTGTAGGTGAGGTTGAT	qRT-PCR primer of Mus *ifn-β*, reverse
GAAATGCCACCTTTTGACAGTG	qRT-PCR primer of Mus *il-1β*, forward
TGGATGCTCTCATCAGGACAG	qRT-PCR primer of Mus *il-1β*, reverse
GTGAACCCCAGACCAGACTG	qRT-PCR primer of Mus *il-18*, forward
CCTGGAACACGTTTCTGAAAGA	qRT-PCR primer of Mus *il-18*, reverse
CTGCAAGAGACTTCCATCCAG	qRT-PCR primer of Mus *il-6*, forward
AGTGGTATAGACAGGTCTGTTGG	qRT-PCR primer of Mus *il-6*, forward
CCTACCTGCTTCTCACCCATACC	qRT-PCR primer of Mus *tnf-α*, forward
TTGATGGCAGAGAGAAGGTTGA	qRT-PCR primer of Mus *tnf-α*, forward

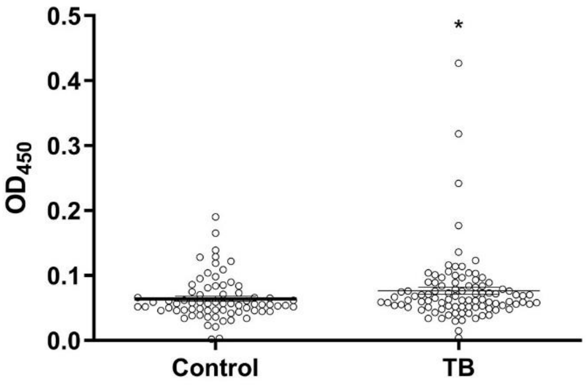

FIGURE S1 ESAT-6-CFP-10 specific antibody in sera of TB patients

Overall IgG level of ESAT-6-CFP-10 fusion protein specific antibody in sera (1:50) of healthy (*n*=72) and TB patients (*n*=96).

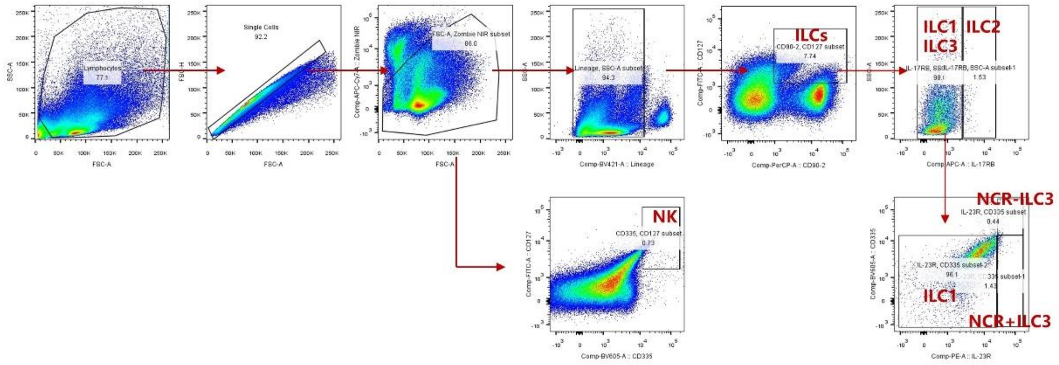

FIGURE S2 Gating strategy for the identification of ILCs in lungs of mice

Representative flow cytometry diagrams depict a gating strategy for counting NK cells, ILC1, ILC2, and ILC3 in lung tissue. ILC3 were further divided into natural cytotoxicity triggering receptor $(NCR)^{+}$ and NCR^{-} subsets. However, further analysis examined the total ILC3 subset due to the lower relative frequency of NCR^{+} cells.
